# Illuminating uveitis: metagenomic deep sequencing identifies common and rare pathogens

**DOI:** 10.1186/s13073-016-0344-6

**Published:** 2016-08-25

**Authors:** Thuy Doan, Michael R. Wilson, Emily D. Crawford, Eric D. Chow, Lillian M. Khan, Kristeene A. Knopp, Brian D. O’Donovan, Dongxiang Xia, Jill K. Hacker, Jay M. Stewart, John A. Gonzales, Nisha R. Acharya, Joseph L. DeRisi

**Affiliations:** 1 Francis I. Proctor Foundation, University of California San Francisco, San Francisco, CA USA; 2 Department of Ophthalmology, University of California San Francisco, San Francisco, CA USA; 3 Department of Biochemistry and Biophysics, University of California San Francisco, San Francisco, CA USA; 4 Department of Neurology, University of California San Francisco, San Francisco, CA USA; 5 Howard Hughes Medical Institute, Chevy Chase, MD USA; 6 California Department of Public Health, Richmond, CA USA

**Keywords:** Metagenomic deep sequencing, Uveitis, Pathogen discovery, Rubella virus

## Abstract

**Background:**

Ocular infections remain a major cause of blindness and morbidity worldwide. While prognosis is dependent on the timing and accuracy of diagnosis, the etiology remains elusive in ~50 % of presumed infectious uveitis cases. The objective of this study is to determine if unbiased metagenomic deep sequencing (MDS) can accurately detect pathogens in intraocular fluid samples of patients with uveitis.

**Methods:**

This is a proof-of-concept study, in which intraocular fluid samples were obtained from five subjects with known diagnoses, and one subject with bilateral chronic uveitis without a known etiology. Samples were subjected to MDS, and results were compared with those from conventional diagnostic tests. Pathogens were identified using a rapid computational pipeline to analyze the non-host sequences obtained from MDS.

**Results:**

Unbiased MDS of intraocular fluid produced results concordant with known diagnoses in subjects with (*n* = 4) and without (*n* = 1) uveitis. Samples positive for *Cryptococcus neoformans*, *Toxoplasma gondii*, and herpes simplex virus 1 as tested by a Clinical Laboratory Improvement Amendments-certified laboratory were correctly identified with MDS. Rubella virus was identified in one case of chronic bilateral idiopathic uveitis. The subject’s strain was most closely related to a German rubella virus strain isolated in 1992, one year before he developed a fever and rash while living in Germany. The pattern and the number of viral identified mutations present in the patient’s strain were consistent with long-term viral replication in the eye.

**Conclusions:**

MDS can identify fungi, parasites, and DNA and RNA viruses in minute volumes of intraocular fluid samples. The identification of chronic intraocular rubella virus infection highlights the eye’s role as a long-term pathogen reservoir, which has implications for virus eradication and emerging global epidemics.

**Electronic supplementary material:**

The online version of this article (doi:10.1186/s13073-016-0344-6) contains supplementary material, which is available to authorized users.

## Background

Ocular infection is an important cause of ocular morbidity and blindness worldwide. However, diagnosis is challenging due to the multitude of possible pathogens. The sensitivity of culture-based assays ranges from 40 to 70 %, and available molecular diagnostic tests target only a fraction of pathogens known to cause ocular disease [1–3]. These limitations are exacerbated by (1) the inability to collect large intraocular fluid volumes given the eye’s small and delicate anatomy, and (2) the difficulty in distinguishing clinically between infectious and non-infectious causes of ocular inflammation.

The urgency to develop better diagnostics for uveitis has been compounded by the recent cases of persistent infection with Ebola virus [4], and possibly Zika virus [5]. These cases highlight the eye’s role as a potential reservoir for infectious agents, with important public health consequences. It is essential that more sensitive, unbiased, and comprehensive approaches are developed to efficiently diagnose ocular infections.

Rapid advances in sequencing technology and bioinformatics have made metagenomics a fertile area for developing clinical diagnostics [6–8]. This prompted us to evaluate a hypothesis-free approach to identify ocular infections by performing unbiased metagenomic deep sequencing (MDS) on clinical intraocular samples from patients with uveitis.

## Methods

### Study design

Six subjects were recruited for a research study using unbiased MDS to identify potential pathogens in intraocular fluid (aqueous or vitreous) (Table 1). This study was conducted according to the guidelines laid down in the Declaration of Helsinki and approved by the Institutional Review Board of the University of California, San Francisco (UCSF). Five of the six subjects served as controls to benchmark the ability of MDS to identify a variety of pathogens; subjects 1–3 had ocular infections with herpes simplex virus 1 (HSV-1), *Cryptococcus neoformans*, and *Toxoplasma gondii*, respectively. HSV-1 and *T. gondii*-directed qualitative PCRs and cultures were performed at the Proctor Foundation, a Clinical Laboratory Improvement Amendments (CLIA)-certified laboratory for ocular testing. Subject 4 had non-infectious uveitis clinically demonstrated by the resolution of intraocular inflammation followed by intraocular injection of a dexamethasone intravitreal implant and the initiation of systemic immunosuppression with antimetabolites. Subject 5 had no ocular inflammation but had intraocular fluid obtained at the time of a retinal membrane peel. MDS was also used to investigate subject 6, who had bilateral uveitis that had defied a 16-year diagnostic work-up at multiple academic centers across two continents (Table 1).

**Table 1:** Results of unbiased metagenomic deep sequencing and conventional diagnostic tests on intraocular fluid samples

Subject	Clinical diagnosis	Sample type	MDS			PCR					RT- PCR	Culture
			Total culture read pairs	Percentage unique non-host read pairs	Organism (number of unique read pairs)	HSV-1	HSV-2	VZV	CMV	*T. gondii*	RV	
1	Anterior uveitis	Aqueous fluid	16,919,211	0.003	*HSV-1* (423)	Pos	Neg	Neg	Neg	Neg	NA	NA
2	Panuveitis	Vitreous fluid	4,551,967	0.10	*C. neoformans* (8469)	Neg	Neg	Neg	Neg	Neg	NA	*C. neoformans*
3	Panuveitis	Vitreous fluid	10,759,511	0.02	*T. gondii* (1853)	Neg	Neg	Neg	Neg	Pos	NA	NA
4	Panuveitis (noninfectious)	Aqueous fluid	9,548,748	0.01	Neg	Neg	Neg	Neg	Neg	Neg	NA	NA
5	Epiretinal membrane (noninflammatory)	Vitreous fluid	7,167,502	0.04	Neg	NA	NA	NA	NA	NA	NA	NA
6	Anterior and intermediate uveitis	Aqueous fluid, right eye	1,684,220	0.41	RV (585)	NA	NA	NA	NA	NA	Pos	NA
		Vitreous fluid, left eye	12,111,540	0.01	RV (10)	Neg	Neg	Neg	Neg	Neg	NA	NA
	Control	H_2_0	983,525	4.13	–	NA	NA	NA	NA	NA	NA	NA

MDS correctly identified known infections in subjects 1–3. Subjects 4 and 5 had non-infectious ocular disease and had negative MDS testing for pathogens. RV was identified via MDS in subject 6 and confirmed by the California Department of Public Health’s RT-PCR assay. Abbreviations: *Pos*, positive; *Neg*, negative; *NA*, not applicable; *RT-PCR*, reverse transcription polymerase chain reaction; *HSV-1*, herpes simplex virus-1; *HSV-2*, herpes simplex virus-2; *VZV*, varicella zoster virus; *CMV*, cytomegalovirus; *T. gondii*, *Toxoplasma gondii*; *RV*, rubella virus; *C. neoformans*, *Cryptococcus neoformans*

### Sequencing library preparation

Samples were prepared for MDS as previously described [6]. RNA was extracted from 20–50 μL of intraocular fluid using TRIzol LS reagent (ThermoFisher Scientific, PA, USA) and the RNA Clean & Concentrator Kit (Zymo Research, CA, USA) per the manufacturers' protocols. Samples were eluted in 20 μL nuclease-free water. Samples were not subjected to DNase treatment. The NuGEN Ovation v.2 Kit (NuGEN, CA, USA) was used to randomly amplify 5 μL of the total extracted RNA to double-stranded complementary DNA (cDNA). cDNA was tagmented with the Nextera DNA Library Prep Kit (Illumina, CA, USA). Depletion of Abundant Sequences by Hybridization (DASH), a novel molecular technique using the clustered regularly interspaced short palindromic repeats (CRISPR)-associated nuclease Cas9 in vitro, selectively depleted human mitochondrial cDNAs from the tagmented library, thus enriching the MDS library for non-human (i.e., microbial) sequences [9]. All samples were subjected to DASH using the same set of single guide RNAs (sgRNAs) as referenced in Gu et al. (2015) [9]. One library was prepared with New England Biolabs’ (NEB) NEBNext RNA First Strand Synthesis Module (E7525) and NEBNext Ultra Directional RNA Second Strand Synthesis Module (E7550) to generate double-stranded cDNA. The cDNA was converted to Illumina libraries using the NEBNext Ultra II DNA Library Prep Kit (E7645) according to the manufacturer’s recommendation and then amplified with 11 PCR cycles. Library size and concentration were determined using the Blue Pippin (Sage Science, MA, USA) and KAPA Universal Quantitative PCR Kit (Kapa Biosystems, Woburn, MA, USA), respectively. Samples were sequenced on an Illumina HiSeq 2500 instrument using 135 nucleotide paired-end sequencing [6, 7]. A water (“no-template”) control was included in each library preparation. Microbial sequences from each sample are located in the National Center for Biotechnology Information (NCBI) Sequence Read Archive [accession ID SRP078679].

### Bioinformatics

Sequencing data were analyzed using a rapid computational pipeline developed by the DeRisi Laboratory to classify MDS reads and identify potential pathogens by comparison to the entire NCBI nucleotide reference database [6]. The pipeline consists of the following steps. First, an initial human-sequence removal step is accomplished by alignment of all paired-end reads to the human reference genome 38 (hg38) and the *Pan troglodytes* genome (panTro4, 2011, UCSC), using the Spliced Transcripts Alignment to a Reference (STAR) aligner (v2.5.1b) [10]. Unaligned reads were quality filtered using PriceSeqFilter [11] with the “-rnf 90” and “-rqf 85 0.98” settings. Reads passing quality control were then subjected to duplicate removal. The remaining reads that were at least 95 % identical were compressed by cd-hit-dup (v4.6.1) [12]. Paired reads were then assessed for complexity by compression with the Lempel-Ziv-Welch algorithm [13]. Read pairs with a compression score <0.45 were subsequently removed. Next, a second phase of human removal was conducted using the very-sensitive-local mode of Bowtie2 (v2.2.4) with the same hg38 and panTro4 references as described above [14]. Read pairs in which both members remained unmapped were then passed on to GSNAPL (v2015-12-31) [15]. At this step, read pairs were aligned to the NCBI nucleotide database (downloaded July 2015, indexed with k = 16mers), and preprocessed to remove known repetitive sequences with RepeatMasker (vOpen-4.0) (www.repeatmasker.org). Finally, reads were aligned to the NCBI non-redundant database (July 2015) using the Rapsearch2 algorithm [16]. On a single 24-core server, processing time varied between 6 and 20 min, depending on the number of non-host reads.

Given the small sample size, we implemented a conservative and simple approach to avoid over-interpretation of the sequencing data. First, the water control was used to identify environmental and laboratory contaminants. The list of organisms detected in the water control was then used to background subtract from the list of organisms detected in the tested patient samples. The remaining organisms were considered to be credible “hits” warranting further confirmatory testing if the following criteria were met: (1) the organism had >20 non-redundant, mapped read pairs per million read pairs (rM) at the species level based on nucleotide alignment, and (2) the organism was known to be potentially pathogenic in the given clinical context of the particular patient.

## Results

### MDS detects pathogens in uveitis

**Fig. 1:**
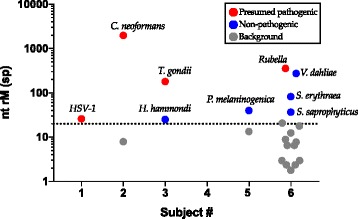
Pathogen identification based on abundance and background subtraction. Organisms in each sample are plotted as a function of matched read pairs per million read pairs (*rM*) at the species level based on nucleotide (*nt*) alignment. For an organism to be considered a potential pathogen, it must have known pathogenic potential and have >20 rM (above dashed line). For subject 3, *H. hammondi* is a eukaryotic organism closely related to *T. gondii.* It is expected that a small fraction of sequences originating from *T. gondii* will align to other closely related organisms. Abbreviations: sp, species; *H. hammondi*, *Hammondia hammondi*; *T. gondii*, *Toxoplasma gondii*; HSV-1, herpes simplex virus-1; *C. neoformans*, *Cryptococcus neoformans*; *P. melaninogenica*, *Prevotella melaninogenica*; *V. dahliae*, *Verticillium dahliae*; *S. erythraea*, *Saccharopolyspora erythraea*; *S. saprophyticus*, *Staphylococcus saprophyticus*

MDS accurately detected viral (HSV-1), fungal (*C. neoformans*), and protozoan (*T. gondii*) infections in subjects 1–3, respectively, and did not detect microbes other than known laboratory and environmental contaminants in subjects 4 and 5 (Table 1). Figure 1 demonstrates that a pre-specified filter of 20 non-redundant rM at the species level effectively eliminated background and reduced the number of potential causative candidates. For subjects 1–3, only the known causative agents passed this filter. Not only did these subjects have confirmatory testing performed in a CLIA-certified clinical laboratory, all three subjects’ clinical courses improved with the appropriate treatment directed at the causative agents. Of note, it is expected that a small fraction of sequences originating from *T. gondii* in the sample from subject 3 will align to other closely related organisms such as *Hammondia hammondi.* The genome coverage for HSV-1 was 9.8 % (14,956 out of 152,222 bases) whereas the total coverage for *T. gondii* was 0.0098 % (62,082 bases out of 62,999,296 bases). Forty-two percent of the *C. neoformans* sequences and 66 % of the *T. gondii* sequences aligned to non-coding regions of their respective genomes, indicating that some genomic DNA was likely sequenced in addition to RNA. Subject 4 was a patient with autoimmune-related panuveitis. His inflammation was controlled with a dexamethasone intravitreal implant, systemic prednisone, and systemic anti-metabolites. The MDS dataset generated from subject 4 contained no pathogen passing our filter (Fig. 1). Subject 5 was a healthy patient who underwent an epiretinal membrane peel and volunteered to donate discarded intraocular fluid for testing. While *Prevotella melaninogenica* had >20 rM in his sample, an infection with this organism was not consistent with this patient’s benign clinical syndrome. Hence, it was considered to be background.

In subject 6, MDS detected a single candidate pathogen: rubella virus (RV) in an aqueous fluid specimen collected in 2014. A total of 585 non-redundant sequence pairs mapped to both the non-structural and structural open reading frames (ORFs) of the RV genome. No sequences aligning to RV were present in the water control or the 18 other cerebrospinal fluid or intraocular fluid samples sequenced on the same run. No RV reads have ever been detected previously in this laboratory.

**Fig. 2:**
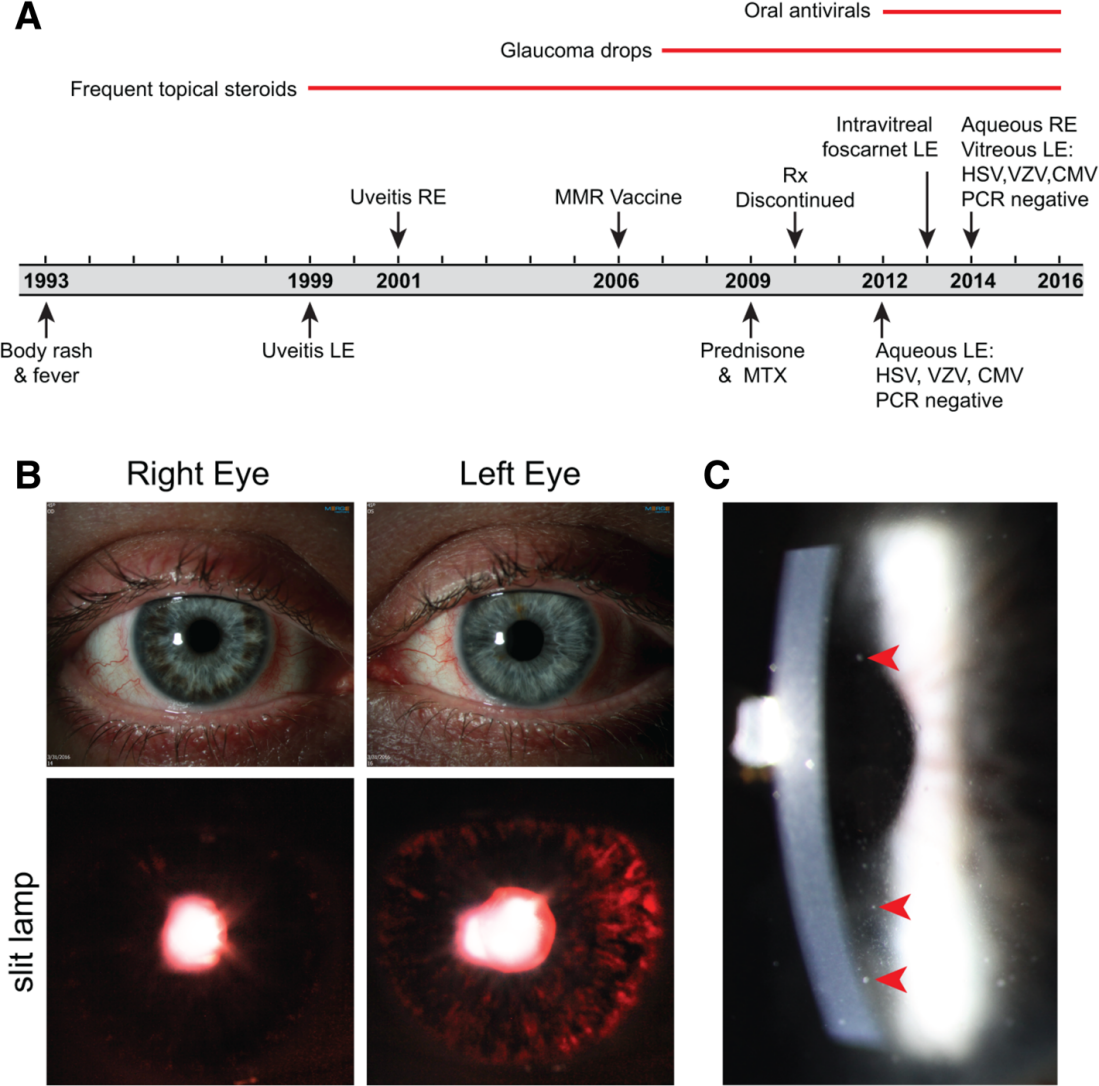
Clinical course and ocular findings of a 40-year-old man with bilateral, idiopathic chronic anterior and intermediate uveitis. **a** Subject 6’s clinical course spanning 22 years. **b** Shows different colored irises (heterochromia) between the right and left eyes (*top panels*) and transillumination defects that are prominent in the left eye because of iris atrophy (*lower panels*). **c** Shows diffused aggregates of inflammatory cells (keratic precipitates; *red arrows*) on the endothelium of the cornea. Abbreviations: *HSV*, herpes simplex virus; *VZV*, varicella zoster virus; *CMV*, cytomegalovirus; *PCR*, polymerase chain reaction; *RE*, right eye; *LE*, left eye; *MMR*, measles/mumps/rubella vaccine; *MTX*, methotrexate; Rx, treatment

Subject 6 was a 40-year-old man with a 16-year history of inflammation in both eyes, whose extensive diagnostic work-up in Germany and the US had not revealed the etiology (Table 1 and Fig. 2a). In 1993 he had a 3-day febrile illness accompanied by a rash that spread from his back to his extremities. He was diagnosed with anterior uveitis of the left eye in 1999, and in 2001 he developed anterior uveitis of the contralateral eye. Topical steroid and non-steroidal anti-inflammatory drops were ineffective. Oral steroids were added in 2009 followed by methotrexate. His inflammation did not improve after 1 year of combined immunotherapy, and his medications were discontinued.

He presented to the Francis I. Proctor Foundation and UCSF in 2012 with moderate anterior and intermediate uveitis associated with ocular hypertension and diffuse stellate keratic precipitates in both eyes (Fig. 2c) and asymmetrical iris atrophy leading to heterochromia (Fig. 2b). These findings were suggestive of viral-related uveitis, and the subject underwent an anterior chamber paracentesis of the left eye. At that time, 100 μL of aqueous fluid was sent for polymerase chain reaction (PCR) testing for cytomegalovirus (CMV), varicella-zoster virus (VZV), and HSV-1/2. Despite negative results, suspicion for viral infection remained high. Antiviral therapy was initiated and continued for 3 years (Fig. 2a), but failed to curb the inflammation. In 2014 he had a paracentesis of the right eye and a therapeutic vitrectomy of the left eye. Repeat infectious disease diagnostics were unrevealing (Fig. 2a).

### Confirmatory testing for RV infection

A 185-nucleotide RNA fragment was reverse transcribed and amplified from subject 6’s aqueous fluid collected from the right eye in 2014, using a published reverse transcription PCR (RT-PCR) assay for detecting the RV E1 gene [17]. Sanger sequencing confirmed that the amplicon was the RV E1 gene (Elim Bio, CA, Hayward, USA). This result was corroborated by the Viral and Rickettsial Disease Laboratory of the California Department of Public Health (CDPH), who performed RT-PCR and Sanger sequenced the 739-nucleotide RV sequence required for genotype assignment (Sequetech Corp., Mountain View, CA, USA) [18, 19]. While the RT-PCR was not quantitative, the level of RV appeared to be low as it was detected at cycle 38. RV was not detected via RT-PCR in nasopharyngeal swab, urine, or tear samples collected in February 2016, indicating that subject 6 was not actively shedding virus. Serologic testing for RV IgG was positive.

An archived sample from the subject’s 2014 left eye vitrectomy subsequently underwent MDS using the same protocol. Although the sample was not flash-frozen and was not stored to optimally preserve RNA integrity, 10 unique sequence pairs aligned to the RV non-structural ORF. While this low number of sequences aligning to RV in the left eye sample did not meet our criteria to be considered a hit, the presence of RV sequences in this sample was considered significant given the known identification of RV in the contralateral eye. The detection of RV in both eyes corroborated the clinical suspicion of bilateral viral infection and demonstrated the robustness of MDS to detect pathogens.

### Characterization of RV sequences

**Fig. 3:**
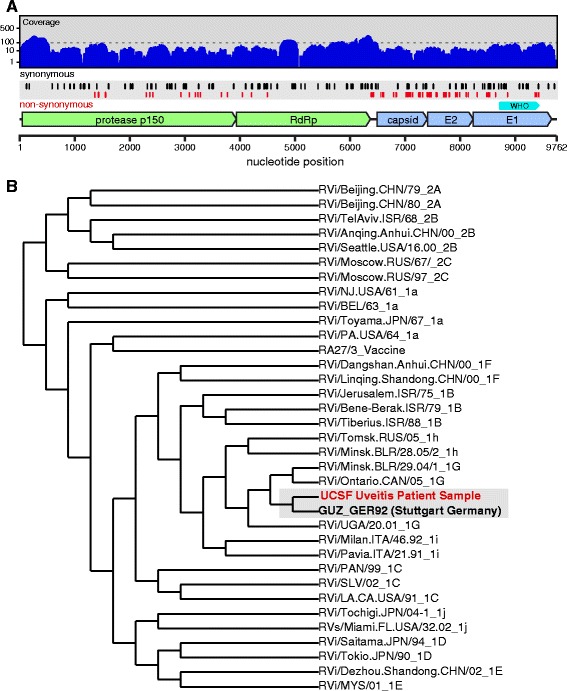
Identification of rubella virus (*RV*) by metagenomic deep sequencing (MDS). **a** Illustrates how the 9688 nucleotide paired-end sequence reads obtained from sequencing the RNA extracted from subject 6’s aqueous fluid aligned to the most closely matched RV genome (GenBank DQ388280.1): 99.3 % of the total RV genome is represented. Positions of synonymous (*black vertical lines*) and non-synonymous (*red vertical lines*) variants are shown. Of the 149 substitutions, 107 were synonymous and 42 were non-synonymous. Of the 42 non-synonymous mutations, 25 occurred within the coding region for the E1 and E2 glycoproteins. Per unit length, the number of non-synonymous mutations in the E1 and E2 proteins was 6.3-fold higher than in the non-structural proteins. The *cyan marker* above the E1 gene represents the 739-nucleotide sequence window recommended by the World Health Organization (*WHO*) for RV genotyping. **b** Phylogenetic analysis of subject 6’s RV strain obtained from MDS with 32 WHO reference strains, GUZ_GER92 (Stuttgart strain), and the RV27/3 vaccine strain, demonstrating that the subject’s RV sequence was most closely related to the genotype 1G viruses and not the vaccine strain

Subject 6’s original MDS data were combined with sequencing data obtained from four replicate sequencing runs. These reads were aligned using bowtie2 v2.2.8 to the complete RV genome (GenBank DQ388280.1) [14]. In total, 9688 base pairs (bp) mapped to the genome, covering 99.3 % of the reference genome (Fig. 3a; GenBank KX291007). This represents the most extensive coverage of an RV genome detected from any intraocular sample and suggests that the RV genomes are full length [20].

### Phylogenetic analysis of the subject’s RV genome

There exists a limited number of complete RV genomes [21] to evaluate the temporal and geographic origins of the RV from this patient. Nevertheless, using the World Health Organization (WHO) classification system for phylogenetic analysis, we found that the patient’s RV strain segregated with the 1G genotype (Fig. 3b). In this analysis, the 739-nucleotide segment of the RV E1 gene isolated from subject 6 with MDS was compared against the 32 WHO RV reference strains using multiple sequence comparison by log-expectation (MUSCLE) [22–24]. Of the three groups seen in the 1G genotype, the group containing the Stuttgart strain circulated in Germany, Italy, and the UK in the early 1990s. Thus, this subject’s RV strain is temporally and geographically most proximate to the RV strain that was known to be circulating when he developed a rash and fever in 1993 in Germany.

The RV sequence (9688 nucleotides) obtained from our subject includes 149 nucleotide substitutions relative to the 1992 Stuttgart strain (GenBank DQ388280.1). This substitution rate of 7.69 × 10^−4^ substitutions/site/year over the 20-year period is within two-fold of the RV evolutionary rate calculated as part of epidemiologic studies evaluating person-to-person transmission (1.19 × 10^−3^ to 1.94 × 10^−3^ substitutions/site/year) [25]. Of the 149 substitutions, 107 were synonymous (Fig. 3a, Additional file 1: Table S1). Of the 42 non-synonymous mutations, 25 occurred within the coding region for the E1 and E2 glycoproteins. Per unit length, the number of non-synonymous mutations in the E1 and E2 structural proteins was 6.3-fold higher than in the non-structural proteins. Considering all mutations in this region, the substitution rate in E1 and E2 was 1.16 × 10^−3^ substitutions/site/year. We note that this mutational imbalance associated with E1 and E2 compared to the non-structural proteins is consistent with persistent viral replication under immunological pressure [21].

## Discussion

MDS correctly identified the causative agent in three infected positive control subjects (1–3). Only environmental contaminants and sequences associated with non-pathogenic organisms were detected in one uninfected subject (patient 5) and one patient with idiopathic uveitis that was likely autoimmune in nature (patient 4). Furthermore, MDS revealed RV in subject 6 who had a 16-year history of idiopathic bilateral uveitis that defied treatment with multiple modalities, including prolonged, systemic immunosuppression. Our results demonstrate that a single unbiased MDS assay can detect fungi, parasites, DNA viruses, and RNA viruses in minute volumes of intraocular fluid from patients with uveitis. The unbiased nature of MDS has potential pitfalls as well. It can be difficult to discriminate between microbes that are present as a result of laboratory or reagent contamination and those that are actually causing disease [26]. For this reason, we have incorporated a simple but useful addition to our analytical pipeline described above that attempts to limit over-interpretation of low abundance microbes identified via MDS that are also present in control samples. Lastly, orthogonal assays like culture, PCR, and serology are still critical for confirmation, as we have highlighted in our cases above.

RV is a positive-sense single-stranded RNA virus in the genus *Rubivirus* of the *Togaviridae* family that causes transient body rash and fever in healthy adults but can also cause devastating birth defects [27]. RV has also been associated with Fuchs uveitis syndrome (FUS), a rare form of chronic intraocular inflammation most often characterized by mild anterior chamber reaction, iris atrophy with or without heterochromia, late-onset ocular hypertension, and minimal associated visual complaints [20, 28–30]. In a subset of patients with FUS, either RV IgG or small fragments of RV RNA have been detected in ocular fluid by Goldmann-Witmer coefficient analysis or RT-PCR, respectively [20, 28, 31]. These tests are only validated for ocular fluid at a few centers in Europe and are not available as clinical diagnostics in the USA.

The protracted diagnostic challenge in our subject was three-fold: (1) diagnostic tests are lacking for ocular inflammation, (2) the subject’s clinical findings were not consistent with FUS until many years after disease onset, and (3) the subject’s relevant infectious exposure occurred 6 years prior to the onset of his ocular symptoms. This case highlights the advantage of a hypothesis-free approach in which a single MDS assay can detect a multitude of pathogens that may or may not have been previously associated with a particular clinical syndrome.

The identification of RV RNA in our subject’s eyes underscores current challenges in infectious disease surveillance and for eradication and elimination programs [32]. The WHO declared RV eliminated in the USA in 2005 as a result of effective and long-standing vaccination policies, but RV remains a threat throughout much of the world [33, 34]. Our subject’s ocular inflammation pre-dated his measles, mumps, and rubella (MMR) vaccination by 7 years, and his RV strain most closely matched the strain circulating in his home country of Germany at the time of his rash and fever in 1993, and not the vaccine strain (Fig. 3b). This is consistent with the notion that RV likely seeded his eyes during this primary infection. Although his immune system cleared the infection peripherally, RV sequestered in the ocular compartment and persisted presumably due to relative immune privilege. Indeed, our analysis of the RV genome provides the first molecular evidence for active RV replication in FUS. Ocular RNA virus sequestration is not a phenomenon relating solely to RV, as Ebola virus was recently detected in the ocular fluid of a patient 9 weeks after resolution of his viremia [4]. Using RT-PCR for RV on our subject’s tears, we were not able to detect shedding of RV, although longitudinal studies are required to determine whether intermittent shedding through tears can occur. As we devise strategies to rapidly identify and control emerging and re-emerging infectious diseases, expanding the scope of pathogen detection to the eyes and other immune privileged sites may be of critical importance.

## Conclusions

Diagnostic tests for intraocular infection fundamentally differ from those for systemic infections because of the small sample volume that can be safely obtained from the eye. Unbiased MDS may circumvent this limitation, as it detects many infectious organisms with a single assay requiring as little as 20 μL of intraocular fluid. Not only does MDS have the potential to alter the paradigm for infectious disease diagnostics in ophthalmology, but it may also provide another valuable public health tool to surveil for re-emerging and emerging infectious diseases in immune privileged body sites.

## Additional file

Additional file 1: Table S1. List of nucleotide substitutions identified in subject 6’s RV genome. The patient’s RV genome was aligned with the Stuttgart strain (GenBank DQ388280.1). A nucleotide change was considered a substitution only if the change was present in ≥4 reads or in 80 % of the total reads at that nucleotide position. (PDF 120 kb)

## Abbreviations

cDNA: Complementary DNA
CDPH: California Department of Public Health
CLIA: Clinical Laboratory Improvement Amendments
CMV: Cytomegalovirus
CRISPR: Clustered regularly interspaced short palindromic repeats
DASH: Depletion of Abundant Sequences by Hybridization
FUS: Fuchs uveitis syndrome
hg38: Human reference genome 38
HSV-1: Herpes simplex virus 1
MDS: Metagenomic deep sequencing
MMR: Measles, mumps, rubella
MUSCLE: Multiple sequence comparison by log-expectation
NCBI: National Center for Biotechnology Information
nt: nucleotide
ORF: Open reading frame
PCR: Polymerase chain reaction
rM: read pairs per million read pairs
RT-PCR: Reverse transcription PCR
RV: Rubella virus
STAR: Spliced transcripts alignment to a reference
UCSF: University of California, San Francisco
VZV: Varicella zoster virus
WHO: World Health Organization

## References

[1] Sugita S, Ogawa M, Shimizu N (2013). Use of a comprehensive polymerase chain reaction system for diagnosis of ocular infectious diseases. Ophthalmology.

[2] Taravati P, Lam D, Van Gelder RN. Role of molecular diagnostics in ocular microbiology. Curr Ophthalmol Rep. 2013;1(4). doi: 10.1007/s40135-013-0025-1. 10.1007/s40135-013-0025-1 PMC3885281 24416712

[3] Endophthalmitis Vitrectomy Study Collaboration. Results of the Endophthalmitis Vitrectomy Study. A randomized trial of immediate vitrectomy and of intravenous antibiotics for the treatment of postoperative bacterial endophthalmitis. Endophthalmitis Vitrectomy Study Group. Arch Ophthalmol. 1995;113(12):1479–96. 7487614

[4] Varkey JB, Shantha JG, Crozier I (2015). Persistence of Ebola virus in ocular fluid during convalescence. N Engl J Med.

[5] de Paula Freitas B, de Oliveira Dias JR, Prazeres J, et al. Ocular findings in infants with microcephaly associated with presumed Zika virus congenital infection in Salvador, Brazil. JAMA Ophthalmol. 2016;134(5):529–535. 10.1001/jamaophthalmol.2016.0267 PMC5444996 26865554

[6] Wilson MR, Shanbhag NM, Reid MJ, et al. Diagnosing *Balamuthia mandrillaris* encephalitis with metagenomic deep sequencing. Ann Neurol. 2015;78(5):722–30. 10.1002/ana.24499 PMC4624031 26290222

[7] Wilson MR, Naccache SN, Samayoa E (2014). Actionable diagnosis of neuroleptospirosis by next-generation sequencing. N Engl J Med.

[8] Pak TR, Kasarskis A (2015). How next-generation sequencing and multiscale data analysis will transform infectious disease management. Clin Infect Dis.

[9] Gu W, Crawford ED, O’Donovan BD, Wilson MR, Chow ED, Retallack H, DeRisi JL (2016). Depletion of Abundant Sequences by Hybridization (DASH): using Cas9 to remove unwanted high-abundance species in sequencing libraries and molecular counting applications. Genome Biol.

[10] Dobin A, Davis CA, Schlesinger F (2013). STAR: ultrafast universal RNA-seq aligner. Bioinformatics.

[11] Ruby JG, Bellare P, Derisi JL (2013). PRICE: software for the targeted assembly of components of (Meta) genomic sequence data. G3 (Bethesda).

[12] Fu L, Niu B, Zhu Z, Wu S, Li W (2012). CD-HIT: accelerated for clustering the next-generation sequencing data. Bioinformatics.

[13] Ziv J, Lempel A (1977). A universal algorithm for sequential data compression. IEEE Trans Inf Theory.

[14] Langmead B, Salzberg SL (2012). Fast gapped-read alignment with Bowtie 2. Nat Methods.

[15] Wu TD, Nacu S (2010). Fast and SNP-tolerant detection of complex variants and splicing in short reads. Bioinformatics.

[16] Zhao Y, Tang H, Ye Y (2012). RAPSearch2: a fast and memory-efficient protein similarity search tool for next-generation sequencing data. Bioinformatics.

[17] Zhu Z, Xu W, Abernathy ES (2007). Comparison of four methods using throat swabs to confirm rubella virus infection. J Clin Microbiol.

[18] Namuwulya P, Abernathy E, Bukenya H (2014). Phylogenetic analysis of rubella viruses identified in Uganda, 2003-2012. J Med Virol.

[19] Standardization of the nomenclature for genetic characteristics of wild-type rubella viruses. Wkly Epidemiol Rec. 2005;80(14):126–32. 15850226

[20] Abernathy E, Peairs RR, Chen MH, Icenogle J, Namdari H (2015). Genomic characterization of a persistent rubella virus from a case of Fuch’ uveitis syndrome in a 73 year old man. J Clin Virol.

[21] Abernathy E, Chen MH, Bera J (2013). Analysis of whole genome sequences of 16 strains of rubella virus from the United States, 1961-2009. Virol J.

[22] Dereeper A, Audic S, Claverie JM, Blanc G (2010). BLAST-EXPLORER helps you building datasets for phylogenetic analysis. BMC Evol Biol.

[23] Dereeper A, Guignon V, Blanc G (2008). Phylogeny.fr: robust phylogenetic analysis for the non-specialist. Nucleic Acids Res.

[24] Edgar RC (2004). MUSCLE: a multiple sequence alignment method with reduced time and space complexity. BMC Bioinforma.

[25] Zhu Z, Rivailler P, Abernathy E (2015). Evolutionary analysis of rubella viruses in mainland China during 2010-2012: endemic circulation of genotype 1E and introductions of genotype 2B. Sci Rep.

[26] Lee D, Das Gupta J, Gaughan C (2012). In-depth investigation of archival and prospectively collected samples reveals no evidence for XMRV infection in prostate cancer. PLoS One.

[27] Lambert N, Strebel P, Orenstein W, Icenogle J, Poland GA (2015). Rubella. Lancet.

[28] Quentin CD, Reiber H (2004). Fuchs heterochromic cyclitis: rubella virus antibodies and genome in aqueous humor. Am J Ophthalmol.

[29] Cunningham ET, Baglivo E (2009). Fuchs heterochromic iridocyclitis--syndrome, disease, or both?. Am J Ophthalmol.

[30] Birnbaum AD, Tessler HH, Schultz KL (2007). Epidemiologic relationship between fuchs heterochromic iridocyclitis and the United States rubella vaccination program. Am J Ophthalmol.

[31] de Groot-Mijnes JD, de Visser L, Rothova A, Schuller M, van Loon AM, Weersink AJ (2006). Rubella virus is associated with fuchs heterochromic iridocyclitis. Am J Ophthalmol.

[32] Dunn G, Klapsa D, Wilton T, Stone L, Minor PD, Martin J (2015). Twenty-eight years of poliovirus replication in an immunodeficient individual: impact on the global polio eradication initiative. PLoS Pathog.

[33] Reef SE, Redd SB, Abernathy E, Kutty P, Icenogle JP (2011). Evidence used to support the achievement and maintenance of elimination of rubella and congenital rubella syndrome in the United States. J Infect Dis.

[34] Pan American Health Organization. Americas region is declared the world’s first to eliminate rubella. 2015. Available from: http://www.paho.org/us/index.php?option=com_content&view=article&id=135%3Aamericas-region-free-of-rubella&Itemid=0&lang=en. Accessed 22 Aug 2016.

