# Erratum to: Illuminating uveitis: metagenomic deep sequencing identifies common and rare pathogens

**DOI:** 10.1186/s13073-016-0377-x

**Published:** 2016-11-22

**Authors:** Thuy Doan, Michael R. Wilson, Emily D. Crawford, Eric D. Chow, Lillian M. Khan, Kristeene A. Knopp, Brian D. O’Donovan, Dongxiang Xia, Jill K. Hacker, Jay M. Stewart, John A. Gonzales, Nisha R. Acharya, Joseph L. DeRisi

**Affiliations:** 1 Francis I. Proctor Foundation, University of California San Francisco, San Francisco, CA USA; 2 Department of Ophthalmology, University of California San Francisco, San Francisco, CA USA; 3 Department of Biochemistry and Biophysics, University of California San Francisco, San Francisco, CA USA; 4 Department of Neurology, University of California San Francisco, San Francisco, CA USA; 5 Howard Hughes Medical Institute, Chevy Chase, MD USA; 6 California Department of Public Health, Richmond, CA USA

## Erratum

It has come to our attention that there is an error in Fig. 3a for this article [1]. The correct version of Fig. 3a can be found below. The red markers now reflect the sequence differences. The text is correct. There was also a row omitted in Additional file 1: Table S1. The revised version can be found below.

**Fig. 3:**
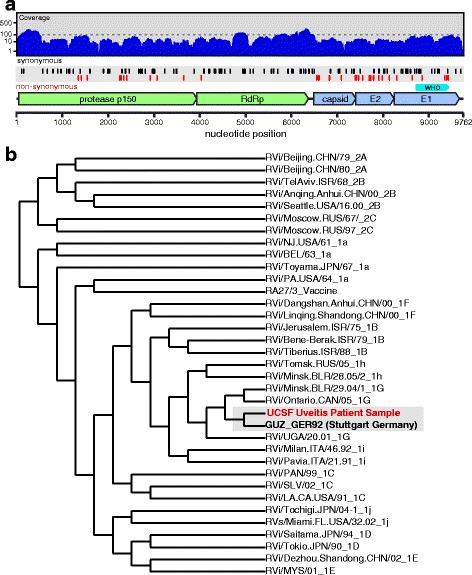
Identification of rubella virus (*RV*) by metagenomic deep sequencing (MDS). **a** Illustrates how the 9688 nucleotide paired-end sequence reads obtained from sequencing the RNA extracted from subject 6’s aqueous fluid aligned to the most closely matched RV genome (GenBank DQ388280.1): 99.3% of the total RV genome is represented. Positions of synonymous (*black vertical lines*) and non-synonymous (*red vertical lines*) variants are shown. Of the 149 substitutions, 107 were synonymous and 42 were non-synonymous. Of the 42 non-synonymous mutations, 25 occurred within the coding region for the E1 and E2 glycoproteins. Per unit length, the number of non-synonymous mutations in the E1 and E2 proteins was 6.3-fold higher than in the non-structural proteins. The *cyan marker* above the E1 gene represents the 739-nucleotide sequence window recommended by the World Health Organization (*WHO*) for RV genotyping. **b** Phylogenetic analysis of subject 6’s RV strain obtained from MDS with 32 WHO reference strains, GUZ_GER92 (Stuttgart strain), and the RV27/3 vaccine strain, demonstrating that the subject’s RV sequence was most closely related to the genotype 1G viruses and not the vaccine strain

## Additional file

Additional file 1: Table S1. List of nucleotide substitutions identified in subject 6’s RV genome. The patient’s RV genome was aligned with the Stuttgart strain (GenBank DQ388280.1). A nucleotide change was considered a substitution only if the change was present in ≥ 4 reads or in 80% of the total reads at that nucleotide position. (PDF 120 kb)
