# Supplementary material for: Erratum to: Illuminating uveitis: metagenomic deep sequencing identifies common and rare pathogens
Source: Genome Med. 2016 Nov 22;8:123. doi: 10.1186/s13073-016-0377-x (PMC5118887; doi:10.1186/s13073-016-0377-x)
Supplement: Additional file 1: Table S1. — List of nucleotide substitutions identified in subject 6’s RV genome. The patient’s RV genome was aligned with the Stuttgart strain (GenBank DQ388280.1). A nucleotide change was considered a substitution only if the change was present in ≥ 4 reads or in 80% of the total reads at that nucleotide position. (PDF 120 kb) [file 13073_2016_377_MOESM1_ESM.pdf]

Nucleotide Position	Nucleotide Change	CDS Position	Codon Change	Amino Acid Change
Non-Structural Protein				
172	C -> T	132	GCC -> GCT	
586	T -> C	546	CGU -> CGC	
685	T -> C	645	CAU -> CAC	
688	C -> T	648	CUC -> CUT	
808	G -> A	768	GGG -> GGA	
907	G -> T	867	GUG -> GUT	
985	C -> T	945	CUC -> CUT	
1,102	G -> C	1,062	GUG -> GUC	
1,156	G -> A	1,116	CUG -> CUA	
1,177	G -> C	1,137	ACG -> ACC	
1,252	C -> T	1,212	AUC -> AUT	
1,362	G -> A	1,322	CGC -> CAC	R -> H
1,399	C -> T	1,359	GCC -> GCT	
1,431	G -> A	1,391	CGC -> CAC	R -> H
1,558	A -> C	1,518	GAA -> GAC	E -> D
1,609	G -> T	1,569	CCG -> CCT	
1,618	C -> T	1,578	UUC -> UUT	
1,912	T -> C	1,872	CGU -> CGC	
2,008	C -> T	1,968	GGC -> GGT	
2,017	A -> G	1,977	GCA -> GCG	
2,053	A -> G	2,013	CCA -> CCG	
2,267	T -> C	2,227	UGC -> CGC	C -> R
2,301	C -> T	2,261	CCG -> CTG	P -> L
2,317	C -> T	2,277	CCC -> CCT	
2,363	T -> C	2,323	UCC -> CCC	S -> P
2,383	C -> T	2,343	CGC -> CGT	
2,404	G -> A	2,364	GAG -> GAA	
2,418	C -> G	2,378	CCG -> CGG	P -> R
2,426	C -> T	2,386	CCC -> TCC	P -> S
2,473	T -> C	2,433	AGU -> AGC	
2,680	A -> G	2,640	ACA -> ACG	
2,719	G -> C	2,679	GCG -> GCC	
2,755	C -> G	2,715	CUC -> CUG	
2,767	A -> G	2,727	GAA -> GAG	
2,833	C -> T	2,793	GUC -> GUT	
2,933	A -> G	2,893	AGC -> GGC	S -> G
2,980	C -> T	2,940	UCC -> UCT	
3,008	C -> A	2,968	CGG -> AGG	

3,031	C -> T	2,991	ACC -> ACT	
3,083	A -> G	3,043	AGC -> GGC	S -> G
3,250	C -> A	3,210	CCC -> CCA	
3,442	A -> G	3,402	ACA -> ACG	
3,445	C -> T	3,405	GGC -> GGT	
3,496	C -> T	3,456	UGC -> UGT	
3,520	T -> C	3,480	GUU -> GUC	
3,666	T -> C	3,626	CUU -> CCU	L -> P
3,892	C -> T	3,852	CGC -> CGT	
4,046	T -> G	4,006	UCA -> GCA	S -> A
4,102	A -> G	4,062	GCA -> GCG	
4,117	C -> T	4,077	ACC -> ACT	
4,216	C -> T	4,176	AUC -> AUT	
4,231	T -> C	4,191	GCU -> GCC	
4,333	C -> G	4,293	GCC -> GCG	
4,348	G -> A	4,308	GCG -> GCA	
4,363	C -> G	4,323	GUC -> GUG	
4,444	A -> G	4,404	UCA -> UCG	
4,453	C -> T	4,413	ACC -> ACT	
4,484	C -> T	4,444	CUG -> TUG	
4,504	C -> T	4,464	UAC -> UAT	
4,570	T -> C	4,530	CUU -> CUC	
4,681	A -> G	4,641	GUA -> GUG	
5,119	C -> T	5,079	GCC -> GCT	
5,188	A -> G	5,148	CAA -> CAG	
5,428	G -> A	5,388	GUG -> GUA	
5,584	A -> G	5,544	CAA -> CAG	
5,593	T -> C	5,553	GUU -> GUC	
5,638	T -> C	5,598	GCU -> GCC	
5,725	C -> T	5,685	AUC -> AUT	
5,944	T -> C	5,904	GAU -> GAC	
5,956	C -> T	5,916	UUC -> UUT	
6,028	C -> T	5,988	AUC -> AUT	
6,058	C -> T	6,018	CCC -> CCT	
6,208	T -> A	6,168	GCU -> GCA	
6,235	T -> A	6,195	GCU -> GCA	
6,286	C -> T	6,246	CUC -> CUT	

Non-coding Region

6,398	C -> T			
6,400	C -> T			
6,405	A -> G			

6,477	A -> G			
6,478	C -> T			

Structural Polyprotein

6,518	T -> C	9	UCU -> UCC	
6,570	C -> T	61	CGC -> TGC	R -> C
6,609	C -> T	100	CCG -> TCG	P -> S
6,628	C -> T	119	CCG -> CTG	P -> L
6,844	G -> A	335	GGG -> GAG	G -> E
6,852	A -> G	343	AGC -> GGC	S -> G
6,869	C -> T	360	CCC -> CCT	
6,875	G -> A	366	CUG -> CUA	
7,046	C -> T	537	ACC -> ACT	
7,055	C -> T	546	CUC -> CUT	
7,076	C -> T	567	GAC -> GAT	
7,114	C -> T	605	CCC -> CTC	P -> L
7,214	C -> T	705	GGC -> GGT	
7,337	C -> T	828	AUC -> AUT	
7,379	C -> T	870	CUC -> CUT	
7,394	C -> T	885	GUC -> GUT	
7,414	T -> C	905	CUC -> CCC	L -> P
7,447	C -> T	938	ACG -> ATG	T -> M
7,450	T -> C	941	CUG -> CCG	L -> P
7,485	G -> A	976	GGC -> AGC	G -> S
7,523	C -> T	1,014	GAC -> GAT	
7,577	C -> G	1,068	GAC -> GAG	D -> E
7,720	C -> T	1,211	CCU -> CTU	P -> L
7,723	T -> C	1,214	UUC -> UCC	F -> S
7,728	A -> T	1,219	ACC -> TCC	T -> S
7,734	G -> A	1,225	GCG -> ACG	A -> T
7,737	A -> G	1,228	AAC -> GAC	N -> D
7,740	A -> G	1,231	ACC -> GCC	T -> A
7,755	A -> G	1,246	ACC -> GCC	T -> A
7,758	C -> T	1,249	CCC -> TCC	P -> S
7,812	T -> G	1,303	UUU -> GUU	F -> V
7,818	T -> C	1,309	UCU -> CCU	S -> P
7,823	G -> A	1,314	GGG -> GGA	
7,925	C -> T	1,416	UGC -> UGT	
7,927	C -> T	1,418	GCU -> GTU	A -> V
7,935	T -> C	1,426	UGG -> CGG	W -> R
7,943	T -> C	1,434	CUU -> CUC	
8,004	G -> A	1,495	GUG -> AUG	V -> M

8,037	C -> T	1,528	CUG -> TUG	
8,060	G -> C	1,551	GCG -> GCC	
8,114	C -> T	1,605	CUC -> CUT	
8,166	G -> A	1,657	GUG -> AUG	V -> M
8,264	C -> G	1,755	GCC -> GCG	
8,326	C -> T	1,817	GCU -> GTU	A -> V
8,420	C -> T	1,911	ACC -> ACT	
8,456	C -> T	1,947	CCC -> CCT	
8,516	C -> T	2,007	GUC -> GUT	
8,531	T -> A	2,022	UCU -> UCA	
8,546	A -> G	2,037	CAA -> CAG	
8,555	T -> G	2,046	UCU -> UCG	
8,567	T -> C	2,058	CCU -> CCC	
8,624	C -> T	2,115	UUC -> UUT	
8,630	C -> T	2,121	CAC -> CAT	
8,659	C -> G	2,150	ACC -> AGC	T -> S
8,711	C -> T	2,202	CAC -> CAT	
8,762	C -> T	2,253	CUC -> CUT	
8,807	C -> T	2,298	UUC -> UUT	
8,819	G -> A	2,310	CCG -> CCA	
8,849	C -> T	2,340	CCC -> CCT	
8,877	A -> T	2,368	AUG -> TUG	M -> L
9,092	C -> T	2,583	GUC -> GUT	
9,152	T -> C	2,643	GCU -> GCC	
9,218	C -> T	2,709	CCC -> CCT	
9,251	A -> G	2,742	GUA -> GUG	
9,375	G -> C	2,866	GGC -> CGC	G -> R
9,414	A -> G	2,905	ACC -> GCC	T -> A
9,434	T -> C	2,925	ACU -> ACC	
9,447	C -> G	2,938	CAA -> GAA	Q -> E
9,620	C -> T	3,111	AUC -> AUT	
Non-coding Region				
9,725	T -> C			
